# An engineering perspective of ceramics applied in dental reconstructions

**DOI:** 10.1590/1678-7757-2022-0421

**Published:** 2023-02-20

**Authors:** Raíssa Monteiro PEREIRA, Renata Guimarães RIBAS, Thaís Larissa do Amaral MONTANHEIRO, Vanessa Modelski SCHATKOSKI, Karla Faquine RODRIGUES, Letícia Terumi KITO, Lucas Kazunori KOBO, Tiago Moreira Bastos CAMPOS, Estevam Augusto BONFANTE, Petra Christine GIERTHMUEHLEN, Frank Akito SPITZNAGEL, Gilmar Patrocínio THIM

**Affiliations:** 1 Instituto Tecnológico de Aeronáutica Laboratório de Plasma e Processos São José dos Campos Brasil Instituto Tecnológico de Aeronáutica (ITA), Laboratório de Plasma e Processos (LPP), São José dos Campos, Brasil; 2 Universidade de São Paulo Faculdade de Odontologia de Bauru Departamento de Prótese e Periodontia Bauru SP Brasil Universidade de São Paulo, Faculdade de Odontologia de Bauru, Departamento de Prótese e Periodontia, Bauru, SP, Brasil.; 3 Heinrich-Heine-University Medical Faculty University Hospital Düsseldorf Düsseldorf Germany Heinrich-Heine-University, Medical Faculty and University Hospital Düsseldorf, Düsseldorf, Department of Prosthodontics, Germany.

**Keywords:** Dental materials, Dental ceramics, All-ceramic, Metal-ceramic, Dental restoration

## Abstract

The demands for dental materials continue to grow, driven by the desire to reach a better performance than currently achieved by the available materials. In the dental restorative ceramic field, the structures evolved from the metal-ceramic systems to highly translucent multilayered zirconia, aiming not only for tailored mechanical properties but also for the aesthetics to mimic natural teeth. Ceramics are widely used in prosthetic dentistry due to their attractive clinical properties, including high strength, biocompatibility, chemical stability, and a good combination of optical properties. Metal-ceramics type has always been the golden standard of dental reconstruction. However, this system lacks aesthetic aspects. For this reason, efforts are made to develop materials that met both the mechanical features necessary for the safe performance of the restoration as well as the aesthetic aspects, aiming for a beautiful smile. In this field, glass and high-strength core ceramics have been highly investigated for applications in dental restoration due to their excellent combination of mechanical properties and translucency. However, since these are recent materials when compared with the metal-ceramic system, many studies are still required to guarantee the quality and longevity of these systems. Therefore, a background on available dental materials properties is a starting point to provoke a discussion on the development of potential alternatives to rehabilitate lost hard and soft tissue structures with ceramic-based tooth and implant-supported reconstructions. This review aims to bring the most recent materials research of the two major categories of ceramic restorations: ceramic-metal system and all-ceramic restorations. The practical aspects are herein presented regarding the evolution and development of materials, technologies applications, strength, color, and aesthetics. A trend was observed to use high-strength core ceramics type due to their ability to be manufactured by CAD/CAM technology. In addition, the impacts of COVID-19 on the market of dental restorative ceramics are presented.

## Introduction

While dental health has substantially improved over the years, it still faces many challenges worldwide. According to a Global Burden of Disease (GBD) study, in 2017, there were approximately 3.5 billion cases of oral conditions, of which 2.3 billion represented untreated caries in permanent teeth, 796 million severe periodontitis, 532 million untreated caries in deciduous teeth, and 267 million had complete edentulism.^1^ These numbers have shown that human habits, such as a sugar-rich diet, are the leading cause of dental caries, and tobacco consumption is a significant cause of periodontal disease.^2 , 3^

Although preventing dental diseases is the preferable way to avoid long, uncomfortable, and expensive dental care, some conditions are inevitable, such as tooth agenesis, tooth loss due to trauma, amelogenesis, dentinogenesis imperfecta, or severe chemotherapy treatment that may negatively impact the quality of the bone that supports the teeth or implants.^4 , 5^ On the other hand, several patients have eagerly sought dental clinics exclusively for aesthetic reasons. As a result of the technological advancements in dental prostheses and restoration materials processes, the market share of dental materials had been projected to reach USD 8.06 billion by 2027, prior to the COVID-19 outbreak.^6^

Due to the pandemic situation, society, the economy, and dental care have suffered unprecedented impacts. In March 2020, the American Dental Association (ADA) proposed virtual meetings for patients and dentists due to the dangerous effects of the virus infection. Additionally, 76% of the clinics were closed, only providing emergency care.^7^

The pandemic exposed the vulnerability of dental care, which requires integration between professionals of various specialty fields. The issue raised by the COVID-19 lockdown could be somewhat circumvented by implementing digital tools and applications. A recent study by Joda, et al.^8^ suggested incorporating the recent digital smart technology into dental medicine. The authors mentioned the top five trends and innovations that can influence the direction of dental research and their stakeholders in the near future. The first one is called rapid prototyping, which can be used for mass production to construct 3D dental models and surgical implant guides quickly and automatically. Another digital tool is augmented and virtual reality, which can be beneficial for prosthetics design allowing for easier communication between the patient and the dentist by using a real-time expanded virtual restoration model. To guarantee a faster and more accurate diagnosis, artificial intelligence (AI) and machine learning are a promising technology for identifying pathologies, predicting disease risk, and proposing therapeutic options. Still on the path of diagnosis, the following application is intended to link individual patient data to population-based citizen groups and biobanks aiming to detect rare diseases and provide novel strategies for research. Specific to dentistry, an AI methodology has been successfully used to predict the debonding of crowns from scanned prepped teeth.^9^ At last, telehealthcare is a complementary tool that does not replace a real dentist, but it may serve to keep self-care in a period of a lockdown due to a pandemic situation. Therefore, considering the advantages, applicability, and obvious limitations, such as emergency care and routine patient procedure, the future direction of dental care should foster the linkage of oral health to the recent digital smart technology.

In dental restorative materials evolution, current developments have been motivated by the quest to mimic tooth features using the biomimetic approach.^10^ To date, the breadth of accomplishments in this field seems to reflect exhaustive efforts to copy teeth’s natural appearance (i.e., aesthetic and optical properties) and, to a much more limited extent, to restore the unique mechanical properties of human teeth.^10^ For a brief description of biomimetics, it is necessary to mention the relationship between the structure and some properties of human teeth. The anatomical structure of human teeth is comprised of an inner structure named pulp that is innervated and highly vascularized, surrounded by a low modulus dentin core (15 – 20 GPa) that is covered by a mineralized high modulus (~70 GPa) layer called enamel.^11 - 16^ Aiming to resemble the color and translucency of natural teeth, dental restorative materials and fabrication techniques are tailored to mimic the anatomical system of dentin and enamel. With proper material selection and case preparation, this goal can be successfully accomplished by dentists working with experienced dental technicians.^12^ The fracture toughness of enamel is approximately ~0.7 MPa.√m in the direction parallel to enamel rods and 1.3 MPa.√m in the perpendicular direction.^17 , 18^ Due to its intrinsic nature, enamel presents a stress-strain response similar to some metals, enabling its function throughout the life of an individual.^19^ Dentin has a fracture toughness ranging from 1 to 2 MPa.√m in the perpendicular and parallel directions to the tubules.^20 , 21^ The complex nature of the dentin-enamel junction (DEJ) has proven to be extremely relevant since it presents a hierarchical microstructure that stops cracks and reduces stresses in enamel as a graded elastic modulus layer.^22^ Detailed work describing the unique structure, micromorphology, and mechanical properties of human teeth and the DEJ can be found elsewhere.^23 , 24^ Ultimately, from a functional biomechanical perspective, human teeth biomimetics makes material development and application techniques challenging to this date. Table 1 shows the values of translucency and mechanical properties of the most currently used dental ceramics.

**Table 1:** Translucency and mechanical properties of representative and commonly used glass ceramics

Ceramic	KIc (MPa.√m)	Translucency parameter (TP)	Biaxial flexural strength (MPa)	E (GPa)	Hardness (GPa)
Lithium disilicate (IPS e.max CAD, Ivoclar Vivadent)^25-27^	1.2 ± 0.26	18 (1.0 mm thickness)	295.8 (1.2 mm thickness)	95	5.8
Zirconia-reinforced lithium silicate (ZLS) CAD/CAM block (Celtra Duo^®^, Dentsply)^28-30^	2.6 ± 0.32	13.3 ± 1.05 (2.0 mm thickness)	105.1 ± 13.70 (2.0 mm thickness)	64	4.5
Zirconia-reinforced lithium silicate – (Vita Suprinity, Vita Zahnfabrik) 25,31	1.2 ± 0.79	31.0 ± 1.0 (1.2 mm thickness)	510 ± 43 (1.2 mm thickness)	70	7
Feldspathic ceramic (Vita Mark II, Vita Zahnfabrik)^32,33^	2.3 ± 0.04	26.4 ± 0.57 (2.0 mm thickness)	112.4 ± 3.2 (1.2 mm thickness)	72	6.2

Although the currently available materials can mimic the gradual change of color and opacity of natural teeth, the microstructure remains a challenge. It would be necessary to build the enamel, DEJ, and dentin the way they are naturally to design a material that resembles human teeth in appearance, mechanical properties, and structural architecture. Ameloblasts form the enamel arranged in a close, overlapping way, forming three zones: inner enamel zone, enamel decussation zone, and enamel parallel prism zone. DEJ is the interphase between enamel and dentin, formed with the alignment of ameloblast and odontoblast with approximately 60–100 μm width of the graded structure. At last, dentin has a structure with tubules that course from the DEJ to the pulp radially inward. Additive manufacturing offers a wide range of possibilities to fabricate materials based upon natural tissues, such as enamel, dentin, and the DEJ, to obtain an overall structure. Therefore, holistic teeth biomimetics remains a challenge for future technologies.^22 , 34^

Among all the available materials, dental ceramics have played an essential role in many fields, such as implants, orthodontics, and prosthodontics.^35^ The preference for ceramics is due to their biocompatibility, aesthetics, durability, and tailored design.^36^ Adding to this, the capacity to have proper translucency, strength, outstanding wear resistance, and intraoral stability make ceramics suitable for routine use in dentistry. In other words, all these properties make this material suitable for various restorative applications.^37^

**Figure 1:**
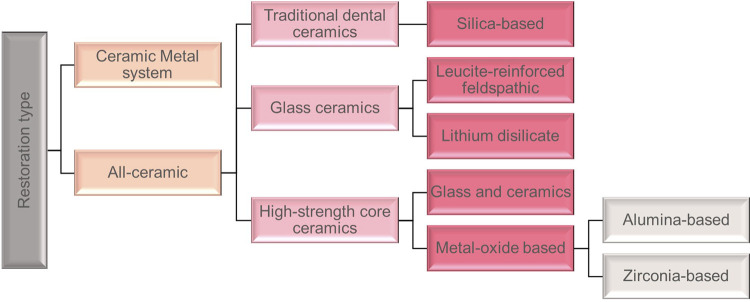
Classification of ceramics restoration type. Adapted from Ho and Matinlinna38

A large variety of ceramics have been used in the dental field according to the restoration type. The two major restoration types are Ceramic Metal System and All-Ceramics, as seen in Figure 1 . Their mechanical and optical properties can be adjusted based on their compositions, type of application, and fabrication methodologies. This study reviews the use of dental ceramics to restore or replace damaged teeth. We will discuss the main factors that influence the performance of the restoration, such as the mechanical properties, fabrication methodologies, design, aesthetic aspects, and applicability.

### Restoration type

#### Ceramic-metal system

Ceramic-metal system, also known as porcelain-fused-to-metal (PFM), was the first configuration used for fixed dental prosthesis fabrication in the early 60s, becoming a well-established treatment approach.^39^ The benefits of these restorations include longevity, strength, and stability of the underlying metal framework that can withstand heavy mastication forces.^40^ PFM is composed of two materials: metal coping and ceramic veneer. Although clinicians advertise all-ceramic systems as a viable option for anterior and posterior Fixed Dental Prostheses (FDP), PFM still presents higher survival rates, especially for implant-supported in long-span prostheses, when compared with porcelain fused to zirconia.^41 , 42^

PFM crowns are widely indicated in oral rehabilitation. The metal alloy core may present several compositions (see ADA dental alloy classification) and is fabricated using different techniques, including casting, subtractive manufacturing or milling, and additive manufacturing or 3D printing.^43 - 45^ Furthermore, the opaque metal substructure is veneered with feldspathic ceramic using conventional and contemporary technologies such as sintering, computer-aided design, computer-aided manufacturing, and heat pressing.^46^

Failure occurrence due to chipping fracture is one of the main concerns when applying PFM restorations. Chipping fracture is usually limited to the veneering ceramic layer due to the lower fracture toughness (K_Ic_) relative to the framework material. One reason for the higher rate of chipping in porcelain fused to zirconia (PFZ), when compared with PFM restorations, is that the virtual absence of the leucite reinforcement in PFZ, tailored to match the coefficient of thermal expansion (CTE) with the zirconia core, results in an approximately 50% decrease in the K_IC_ of the veneering porcelain.^47^ When chipping occurs, the core material usually remains unexposed since it is covered with a thin veneer ceramic layer on its surface. Also, the CTE mismatch between the veneering and the framework material is an essential source of residual stresses. This mismatch can be beneficial when framework’s CTE is higher than veneer’s CTE. If metal contracts slightly more than the porcelain upon cooling from firing to room temperature, it leaves the porcelain in residual compression. The propagation of potential cracks in the porcelain material is suppressed by these compressive stresses, increasing the ceramic fracture resistance.^48^Table 2 shows some of the values of CTE of the metal framework and ceramic veneer.

**Table 2:** CTE values of material framework and ceramics veneer

Material	CTE (ppm K−1)
Cobalt–Chromium alloy	14.1 (49)
Nickel–Chromium alloy	14.8 (49)
Zirconia 3Y-TZP	10.5 (50)
Porcelain VM9	9.2 (51)
Porcelain VM13	13.4 (51)

Tanaka, et al.^52^ (2019) evaluated the residual thermal stresses via edge chipping resistance of PFM and veneered zirconia structures. The two groups of bilayer bar specimens were prepared with zirconia (Y-TZP) and Ni-Cr alloy veneered with commercial porcelains, VM9 and VM13, respectively, with a final thickness of 1.5 mm. Each group of samples was subjected to a different cooling protocol – fast and slow. The chipping resistance was measured using the edge chipping method, where the load was applied in two directions, parallel and perpendicular to the veneer/framework interface. The results showed that the PFM and PFZ specimens had different chipping resistance. The slowly cooled veneered zirconia presented significantly higher chipping resistance (251 N/mm) than the PFM (163.3 N/mm), considering a perpendicular load. This result for PFM can be related to the thermal effects on the NiCr alloy. For the K_Ic_, it was observed that PFM fast cooling and PFZ slow cooling with a perpendicular load presented the highest value (1.2 MPa.√m). This value is attributed to the residual compressive stresses that can increase the load necessary to initiate the median cracks or modify the crack growth velocity.

The manufacturing method can also influence material properties. Zhou, et al.^53^ (2019) produced Co-Cr-based devices for dental restoration using two different techniques, selective laser melting (SLM) and cast alloy (CAST), to evaluate the bond strength between the metal framework and the ceramic veneering. The Co-Cr-based devices obtained from each technique were covered, by fusion, with a thin layer of opaque porcelain followed by body porcelain. CTE tests and 3-point bend tests evaluated the bond properties. Three-point flexural results showed that the bond strengths of SLM specimens (45.8 MPa) were significantly lower than that of CAST specimens (54 MPa). The surface analysis of SLM specimens revealed a mixed fracture type of adhesive, cohesive fractures, and lower area fractions of porcelain adherence. On the other side, the CTE results presented similar values for both techniques, $14\times 10^{-6}$ ºC^-1^ for the CAST group and $14.5\times 10^{-6}$ C^-1^ for the SLM group.

Given these results, it was suggested that the CAST method presented the best dynamic combination of CTE with the porcelain. Based on the mechanical characterization and microstructural evaluation, the CAST group was better than the SLM group.

Considering the aesthetic aspect, an ideal restoration should match the contour, color, surface texture, fluorescence, translucency, and opalescence of natural teeth.^55^ Metal-ceramic restorations have been extensively used in restorative dentistry due to their high fracture strength. It still represents the material with the widest indications in oral rehabilitation and has a precise use to mask darkened substrates such as titanium implant abutments or darkened teeth.^12^ However, the metal substructure prevents light transmission and makes it challenging to achieve an acceptable masking effect.^12 , 55^

## All ceramic

### Traditional dental ceramics

*Silica-based ceramics*

Silica-based ceramics were the first materials used in dentistry to make porcelain prostheses.^56^ Also called feldspathic ceramics, this composition belongs to traditional ceramic materials widely used in all-ceramic restoration. This material is classified as porcelain-based because it undergoes a vitrification process in which numerous crystalline cores are surrounded by a silica-based glassy matrix.^57^ Such microstructure is formed due to the high temperature used to process the raw material composed of silica-based ceramics.

The main composition of this traditional dental ceramics is 70 – 75% of potash feldspar ( $K_{2}O\cdot {Al}_{2}O_{3}\cdot 6{SiO}_{2}$ ), 15 – 20% of quartz (SiO_2_) as crystalline phase, and the remainder of kaolin ( ${Al}_{2}O_{3}\cdot 2{SiO}_{2}\cdot 2H_{2}O$ ) as a binder.^58^ Since quartz is not a strong material, Al_2_O_3_ is added to improve mechanical performance.^57^ Also, a few amounts of pigments can be used to reach different ranges of opacity and translucency, producing different tooth shades.^59^

Silica-based ceramics can mimic the shades of a natural tooth, making them suitable for veneer inlays and onlay restorations.^38^ However, this type of ceramic restoration is very brittle, which makes them suitable only for low-stress-bearing anterior applications.^60^

The application of silica-based ceramics as a veneer is made by a mixture of fine glass particles, around 25 µm diameter, with polymeric binders in an aqueous medium to form a powder slurry.^61^ This solution can be applied directly on a dental core fabricated by metal, called PFM, or another ceramic core, such as zirconia or lithium disilicate.^62^ The porcelain layer is heated slowly to evaporate the binder and to coalesce the particle to form a dense part, and then it is cooled slowly to prevent cracking and crazing.^61^

Despite the outstanding customizable aesthetics, the bilayered structure may present, the strong ceramic core coated with porcelain faces some drawbacks regarding properties incompatibility. Chipping and delamination are the main causes of failure due to, in part, the weak bonding between the core and veneer.^52 , 63^ Usually, the porcelain layer has lower toughness and CTE.^64^ In addition, the multiple steps involved in the veneering process develop residual stress that negatively affects the adhesion of the porcelain on the core surface.^65^ Hence, an alternative approach is the monolithic ceramic design fabricated directly by CAD/CAM or press technology (e.g., glass ceramics).^66^

There are many ways to evaluate the aesthetic aspect of fabricated ceramic teeth. One of them is the visual method, in which the natural tooth is compared with a shade guide provided by the manufacturer. The most used shade guide is Vita Classical from Vita Zahnfabrik, which presents the appearance of the samples arranged by groups according to the hue (A1-D4).^67^ However, each porcelain system can only precisely match in hue with its proprietary guide system.^68 , 69^ Another way to evaluate the aesthetic aspect is by the masking ability of the dental material, which can be defined as the color difference result (ΔE) between the core and the coating.^70^ When this value equals zero, the background color is hidden by the outlying structure. A parameter that influences the masking ability is opalescence, which is one of the optical properties that displays the blueness of the reflected light spectrum and brownness-orangeness of the transmitted light spectrum. Opalescence is affected by the material composition, particle size, and thickness of the ceramic.^71^

To report the relationship between layer size and optical properties, Valizadeh, et al *.* ^72^ (2020) investigated the effect of ceramic thickness on opalescence parameters. The feldspathic ceramic was compared to lithium disilicate and zirconia 3^rd^ generation. Cylindrical samples of 10 mm diameter with 0.5 and 1.0 mm thick of feldspathic ceramic samples were produced using an aqueous solution of porcelain powder followed by baking under vacuum to a maximum of 920 °C. Lithium disilicate samples were prepared by the wax removable die technique. Lastly, zirconia 3^rd^ generation was obtained by machining monolithic blocks. A spectroradiometer measured the opalescence in the transmittance and reflectance modes. The effect of ceramic type on opalescence was significant, while the effect of ceramic thickness on opalescence was not. Also, the interaction effect between ceramic type and ceramic thickness on opalescence was significant. The results showed that in all ceramic groups, except for lithium disilicate, the mean opalescence of 1 mm thickness was higher than that of 0.5 mm thickness specimens. This was expected since higher thicknesses allow light transmission through the media resulting in incomplete masking and increased opalescence. Usually, a ceramic restoration comprises an opalescent material, ceramic, an A2 shade, and a masking agent. Thus, the discussion pointed out that the reason for the opalescence increase with higher thickness was the composition of each material. Since lower amounts of masking agent promote a higher share of the opalescent agent in the scattering of blue light, lithium disilicate, which has a low masking agent, presented decreased opalescence with 1 mm thickness. Alternatively, feldspathic ceramic has a limited amount of opalescent material in its composition, making it the most translucent ceramic among the tested groups.

Staining of ceramic restorations is a procedure widely used to mimic the nuances and shades of natural teeth. This occurs because monolithic restorations without subsequent customization after milling do not meet high aesthetic demands. Unfortunately, the stain layer is removed by wear processes. To understand which ceramic material allows the maintenance of the staining layer for a more extended period, Dal Piva, et al.^73^ (2021) studied the toothbrushing wear resistance of stained CAD/CAM ceramics, including the feldspathic type. The samples were obtained from a precisely cut block with the dimensions of 10×8×6 mm. Then, the specimens were directly stained. The specimens were brushed for 150,000 cycles at 2.45 N with 180 strokes/min. Mean roughness depth (Rz) surface measurements were performed after 50,000, 100,000, and 150,000 cycles, corresponding to 5, 10, and 15 years of toothbrushing in the oral environment. The results were compared with other stained ceramic systems, such as high-translucency zirconia, zirconia-reinforced lithium silicate, and hybrid ceramic. After the three measurement occasions (5, 10, and 15 years), no difference was observed between the simulations, except for the hybrid ceramic. The feldspathic ceramic presented a higher wear resistance, showing its superior longevity compared with other ceramic types.

Silica-based ceramics coating can create definitive restorations with individualized and natural optical characteristics. In contrast, this type of ceramic presents a lower survival rate that limits its application to the anterior region. Thus, silica-based ceramics are suitable for single-tooth restorations, such as veneers, inlays, and onlays.

## Glass ceramics

### Leucite reinforced feldspathic ceramics

Leucite is a potassium aluminum silicate with a composition of tetragonal KAlSi_2_O_6_, morphology of lamina-like crystals, and size from 1 to 5 µm.^74^ At high temperatures, leucite exists as a cubic polymorph.^75^ Leucite-based glass-ceramic can be commercially found as IPS Empress® and Finesse®, among others, usually manufactured by hot press technology or CAD/CAM.^74^

Leucite crystals reinforce the glass by restricting and defecting the propagation of cracks. This glass ceramic presents a high CTE, attractive aesthetics due to high and adjustable translucency, and the possibility of coloring the glass by adding metal oxide pigments.^61^ Their strength is almost double the strength of traditional feldspathic porcelains but not enough for use as posterior fixed dental prostheses.^61^ Leucite-based materials can be clinically applied as resin-bonded laminate veneers, inlays, onlays, and anterior and posterior crowns.^76^

An efficient way to increase the coefficient of thermal expansion of feldspathic glasses is by adding crystals of tetragonal leucite.^61^ When dental porcelain reinforced with leucite is cooled, leucite suffers phase transformation from cubic to tetragonal, which causes a contraction along the α axis, added to the contraction from cooling. Due to the resultant contraction and the significant difference between the CTE from leucite and the glassy matrix, tangential compressive stress is formed around leucite particles, which is responsible for strengthening feldspathic dental porcelains. This stress may also produce microcracks, which will disaggregate the leucite particles from the matrix and promote the propagation of cracks. Despite the ability to toughen glassy matrices, leucite is inherently brittle and cannot deflect a crack. The fracture toughness was improved by about 60% and flexural strength by about 36% after the introduction of leucite.^77^

Vasiliu, et al.^78^ (2020) studied the effect of thermocycling, surface treatments, and microstructure on the properties of heat-pressed glass-ceramic with 50% of leucite (Vita PM9). Ceramic ingots with a thickness of 1.5 mm were prepared according to the manufacturer’s instructions and were glazed on one side and polished on the other. Thermocycling was performed in distilled water to estimate 10 years of oral conditions (10,000 cycles). The translucency parameter was reduced after the thermocycling, and the opalescence values were increased, both for glazed and polished surfaces. The same behavior was observed for Rz and Ra (arithmetical mean roughness), which were higher after aging for both surfaces. The glazed surfaces were more affected after thermocycling, showing higher roughness and suggesting that the glaze layer is degraded first during aging. Before thermocycling, the surfaces were uniform, with leucite crystals ranging from 10 – 20 µm, and easily identified. The glazed surface presented round-shaped forms. After aging, the leucite crystals could not be distinguished from the matrix, and the glazed surface suffered several cracks. These changes in morphology are attributed to the large grain size of feldspathic ceramics, between 2 and 4 µm.

Gönüldaş, et al.^79^ (2019) evaluated the influence of surface finishing on leucite-reinforced ceramics (IPS Empress CAD^®^). One group of machinable leucite-reinforced glass-ceramic was manually polished, and the other group was glazed using liquid glaze and fired according to the manufacturer’s recommendations. The finishing technique caused statistically significant differences in the surface roughness; so that polished surfaces presented lower roughness than glazed ones. The translucency parameter was higher for polished samples; however, the difference was not significant when compared with the glazed group. Concerning flexural strength, polished samples presented an average value of 82.83 MPa, whereas glazed samples presented a mean value of 152.87 MPa. The authors concluded that polishing is more effective than glazing, for surface roughness; however, glazing increases the flexural strength of the leucite-reinforced glass ceramics.

In conclusion, the leucite-reinforced feldspathic ceramic has higher CTE values. This property decreases the thermal mismatch between the ceramic and the metal when using the PFM configuration. Leucite-reinforced porcelain also has a CAD/CAM block version.

## Lithium disilicate ceramics

Lithium disilicate (LDS) is a glass-ceramic widely used in dental applications due to its good mechanical properties, biocompatibility, and aesthetic performance. LDS is composed of 65 vol% of lithium disilicate (Li_2_Si_2_O_5_) with small needle-shaped crystals embedded in a glass matrix with 1 vol% porosity. These features make LDS one of the most popular all-ceramic materials for dental restorations.^80^

The good mechanical properties of this glass-ceramic are due to two major factors: 1) their elongated disilicate crystals form an interlocking pattern, which hinders crack propagation, and 2) the divergence between the thermal expansion coefficients of the crystalline and glass phases, which induces compressive stress around the crystals. Moreover, LDS shows good biocompatibility, particularly with soft tissue, because of its silica content. *In vitro,* this material presents adhesion and proliferation of human epithelial cells and gingival fibroblasts, especially on polished surfaces.^81 , 82^ LDS restorations presented no inflammatory reactions *in vivo* and are beneficial for the natural and healthy aspect of soft tissues when in contact with marginal gingiva or peri-implant mucosa. Furthermore, LDS presents excellent translucency, around 30% higher than conventional zirconia (3Y-TZP) containing ≤1.0 wt% of (Al_2_O_3_).^82^ Conversely, some disadvantages can limit their utilization, such as abrasiveness and wear, which are highly dependent on the surface characteristics of the restoration.

Chemical stability, translucency, good flexural strength, and K_Ic_ are some of the properties that make this glass-ceramic so popular and versatile, permitting LDS to be suitable for posterior areas in different applications, such as anterior veneers and posterior inlays, onlays or overlays, 3-unit FDP (premolar region) tooth- and implant-supported single crowns (SC), and bilayered and monolithic restorations.^83^ The blocks used in monolithic restorations are available in different colors and translucency, depending on the ions or size and distribution of the crystals dispersed on the glassy matrix, respectively.

Studies concerning controlling translucency and mechanical properties of LDS ceramics have been done in the past few years. Jung, et al.^84^ (2021) demonstrated a control method for tailoring the translucency of LDS glass ceramics through thermal refinement. The results of LDS microstructure and translucency for four different heating treatment schedules were compared. Microstructure plays a vital role in ceramics translucency, which can be controlled by changing the volume, size, and density of crystals. The high temperature decreases glass phase viscosity and raises the mobility of molecules in ceramics, which facilitates crystal growth while holding time enhances the number of crystallites. The results showed that the higher the crystal density, the lower the ceramic translucency, due to less light scattering. However, the particle size should be small enough to achieve minimal grain-boundary light scattering. Besides, the heat treatment can modify the percentage of particles that allows visible light transmission.

The mechanical strength of the current dental products of LDS is not adequate for some specific dental applications.^85^ Heat-treatment temperature and holding time can also be an alternative to modify the mechanical properties of LDS glass ceramics. Sun, et al.^86^ (2021) investigated the influence of the three-stage heat treatment on microstructure and mechanical strength. The results showed that the lithium metasilicate (LMS) phase is initially formed at 619 °C, then decomposed and recrystallizes as the LDS phase at 789 C. At the third stage (850 ^o^C, holding time of 3 h), the morphology of LDS crystals was modified from spherical to rod-shaped. The bending strength was significantly enhanced for the glass-ceramic with a large amount of uniform round-shaped LDS crystals (325 MPa). The results also suggest that the increased temperature of the second stage could be beneficial to the transition from LMS to LDS crystals. For LDS glass ceramics, the major fracture type is intergranular since the glass matrix is more brittle and has lower tensile resistance than the crystalline phase. The fractures will occur in the weaker residual glass, between the crystals and the glass, and propagate through the glass matrix. The fracture surfaces demonstrate that LDS crystals can retard the propagation of cracks in the samples. Thus, heat-treatment can be an alternative to change some properties, such as mechanical strength and translucency.

Long-term (10 to 16.9 years) clinical retrospective data on the performance of LDS inlays and onlays, crowns, and partial crowns as a function of multiple variables have been published, and cumulative survival rates are all above 90%.^87 , 88^ However, although high survival rates, comparable to PFM, were observed for LDS monolithic FDP at 10 years (87.9%), an impressive decrease to only 48.6% in survival was reported at the 15-year follow-up, suggesting a decisive role of fatigue and crack propagation over time.^89 , 90^

Due to its good mechanical, optical, aesthetics, and biological properties, combined with reduced thickness, favorable wear behavior, and minimally invasive approach, LDS is currently one of the most popular metal-free materials for dental restorations. New lithium disilicate materials have been launched in the market with essential innovations in the glass composition and crystal structure.^91^ However, some drawbacks can limit their use in dental applications. A different approach has been developed to overcome these limitations.

In addition to leucite-reinforced feldspathic ceramic and LDS classified as glass ceramics, there is zirconium-lithium silicate (ZLS). ZLS is an alternative approach for the optimized CAD/CAM technology, and it is composed of a lithium silicate ceramic reinforced with 10% zirconia, thus achieving optimized translucency and high durability. Its microstructure is formed by a homogeneous glassy matrix containing a crystalline component of round and submicrometric elongated grains of lithium metasilicates and orthophosphates. Additionally, it contains tetragonal zirconia to increase strength value.^92 , 93^ Hence, according to the manufacturer’s instructions, ZLS is indicated for anterior and posterior crowns, veneers, inlays, and onlays.^94^

## High-strength core ceramics

### Metal-Oxide Based Ceramics

*Alumina-Based Ceramic*

Alumina, or aluminum oxide (Al_2_O_3_), is a chemically inert ceramic with clinical applications based on its biocompatibility, density, strength, and wear resistance.^95^ In Dentistry, high-density and high-purity alumina ceramics (>99.5%) were used in dental implants due to excellent corrosion resistance, good biocompatibility, high wear resistance, and moderate mechanical strength. Alumina has been used in dental applications for fabricating copings and frameworks of full-coverage crowns and fixed prostheses.^96^ Currently, polycrystalline alumina is no longer available.

Significant progress has been made to overcome the inherently brittle nature of Al_2_O_3_.^97^ The mechanical properties of alumina were improved by the addition of ZrO_2_ as a reinforcing agent, creating the zirconia toughened alumina (ZTA), a polycrystalline ceramic composite of alumina matrix and a disperse phase of metastable tetragonal zirconia.^98^ The enhancement of K_Ic_ in the ZTA composite is mainly attributed to the stress-induced phase transformation that tetragonal (t) zirconia undergoes into the more stable monoclinic (m) phase.^97^ In addition, the transformation t → m can generate a state of compression in the alumina matrix due to the volumetric expansion of ZrO_2_ (3 - 5%).^99^

The mechanical properties of ZTA can be controlled by changing the ZrO_2_ content for specific applications. For example, Hu, et al.^97^ (2020) investigated the effects of the Y_2_O_3_-stabilized ZrO_2_ content (from 0 wt% up to 41.5 wt%) on the mechanical properties of ZTA composites. Mechanical tests showed that an increase of ZrO_2_ content (over 20 wt%) decreased microhardness, whereas K_Ic_ increased. However, the limitation of using ZrO_2_ is related to a slow transformation from tetragonal to monoclinic phase (t → m), which occurs in a humid atmosphere, followed by microcracking and a loss in strength in a phenomenon called low-temperature degradation (LTD). Thus, several studies have been carried out to predict the aging behavior of ZTA composites.^100^

A recent study carried out by Jalkh, et al.^101^ (2020) evaluated the effect of aging in a composite of alumina matrix reinforced by 30 wt% of 3Y-TZP and compared the results with samples prepared with high-purity alumina (particle size of 350 nm) and pure 3Y-TZP (Zpex; particle size of 40 nm). High-density samples (up to 95%) were prepared by uniaxially pressing and were subjected to an artificial aging (20 h, 134 °C, 0.22 MPa). They observed that the optical and mechanical properties of the ZTA-Zpex composite and alumina remained stable after artificial aging, while 3Y-TZP was affected by the aging process. A higher contrast ratio (CR=0.99) and lower translucency parameter (TP=0,42) values were obtained for the ZTA-Zpex composite relative to the alumina (CR=0.95 and TP=2.53). XRD analysis of ZTA-Zpex revealed the stability of the tetragonal phase of the composite after aging showing a slight increase in the monoclinic content from 1.3% to 3.3%, before and after autoclave aging, respectively. On the other hand, 3Y-TZP showed a considerably increase in the amount of monoclinic zirconia after aging (21.9%). Therefore, once the t → m phase transformation occurs to a small extent in ZTA-Zpex composites, the K_Ic_ significantly increased (∼910 MPa), when compared with pure alumina (∼410 MPa), not showing a significant difference after artificial aging. The improvement in mechanical performance was related to stress-induced phase transformation. The toughening mechanism prevents crack propagation and deflection due to the residual compressive stress of the thermal expansion incompatibility of the alumina and 3Y-TZP.

Today, a reliable implant should exhibit more than 30 years of lifetime. In contrast, the lifetime of ZTA is around 10 years, which exposes a field of opportunity for future improvements.^102^ Recent studies have focused on long-lasting devices based on new materials characterized by superior strength and toughness, optimal tribological properties, and long-term biocompatibility. While alumina provides high strength and hardness, tetragonal zirconia exerts a toughening effect. Thus, exploiting alumina-zirconia composite ceramic, as an alternative to monolithic alumina and zirconia, seems to be the path for enhancing mechanical properties. Further studies on improving different techniques for preparing dense ceramics are still necessary.

*Zirconia-based ceramics*

Zirconium dioxide (ZrO_2_), also known as zirconia, has been the most promising material in dental restorations with several advantages, including superior flexural strength, K_Ic_, biocompatibility, biofunctionality, and affordability.^103^ Advanced zirconia-based ceramics are widely used in oral rehabilitation as prostheses to replace the unit, and partial and total absences on teeth and implants.^36^

ZrO_2_ presents polymorphism and can exist in three different crystallographic arrangements as the temperature changes – monoclinic, tetragonal, and cubic.^100^ The high K_Ic_ (3 − 5 MPa√m) of this material is explained by the tetragonal phase transforming into a monoclinic phase, leading to a compressive stress field around the tip of microcracks present in ceramic. This prevents the crack from growing, and the material gains more resistance against failure propagation.^104^ Moreover, when the material presents a high tetragonal phase content, the flexural strength exhibits 1000 − 1200 MPa.^105^

During the ZrO_2_ development, four different generations have been developed, seeking improvements to reach better optical and mechanical properties. From the description of the generations’ developments, a trade-off between aesthetics and mechanical properties was clearly evident for some generations. The first two generations of zirconia were composed of 3 mol% of Y_2_O_3_ (cubic phase stabilizer), but their optical properties needed improvement to reach the aesthetic demands due to the amount of alumina in its composition (approximately 0.25 wt.%). This aesthetic limitation drove the use of 1^st^ generation zirconia in prosthetic infrastructures requiring the veneer with traditional feldspathic porcelain to achieve a more natural look.^106^

The 2^nd^ generation of zirconia reduced five times the alumina content and changed the sintering temperatures and cycles to control particle sizes and microstructures, possibly improving their optical properties. However, its application as monolithic crowns (without porcelain) still resulted in unsatisfactory aesthetic properties, limiting its use for posterior areas of the mouth.^107^ The positive aesthetic effects of these restorations were still inferior to lithium disilicate and leucite-reinforced ceramics. This led to the development of new translucent varieties of zirconia, aiming to improve their transmittance to be used in aesthetically demanding clinical situations.^108^

**Figure 2:**
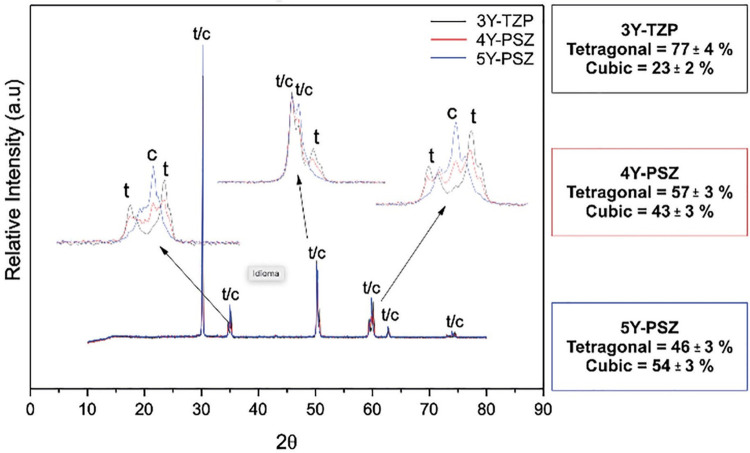
XRD of zirconia 3Y-TZP 2nd generation and zirconia 4Y/5Y-PSZ 3rd generation illustrating in the rectangle the quantification of the tetragonal and cubic phases according to Rietveld’s analysis114; Attribution 4.0 International (CC BY 4.0)

Due to this limitation, the 3^rd^ generation of zirconia increased the amount of Y_2_O_3_ to 4 – 5 mol% aiming to increase the ratio of cubic phase.^109^ This strategy was used because the cubic microstructure allows a better light transmission regarding its larger grains and isotropic geometry, while significantly compromising mechanical properties compared to the 1^st^ and 2^nd^ generations.^110^ Ultimately, from a clinical standpoint, although the 3^rd^ generation of ultra-highly translucent zirconia is suitable for a single monolithic unit or posterior prosthesis, lithium disilicate glass-based ceramics have dominated the market since they are still recognized as materials with more predictable aesthetic outcomes.^111 , 112^ Also, long-term (≥10 years) high survival rates have been reported for lithium disilicate, monolithic or bilayered, in anterior and posterior restorations.^87 , 88 , 113^Figure 2 shows the XRD spectra and quantification of the crystalline phases of the three types of zirconia. From this, we can infer that more quantity of yttria stabilizer generates more content of cubic phase. Thus, translucent zirconia has higher amounts of cubic phase and, consequently, less amount of tetragonal phase (c-phase: 5Y-PSZ>4Y-PSZ>3Y-TZP).^114^

The current development of a novel ZrO_2_ generation, which we call the 4^th^ generation, comprises a multilayer system. This new generation was developed in an attempt to mimic the gradual change of color and translucency of the natural teeth without compromising the mechanical properties. The multilayer system works with different amounts of cubic phase in each layer.^115^ However, this phase is also known for its lower mechanical properties compared to the tetragonal phase. Thus, the challenge now is to understand the mechanical behavior of this new system by studying the complex interface between the layers. Table 3 shows the composition, material properties, and application of each generation of zirconia.

**Table 3:** Composition, material properties, and application of zirconia66,116

Composition	3Y-TZP	3Y-TZP	4Y/5Y-TZP	Multilayer
	**1** ^**st**^ **generation**	**2** ^**nd**^ **generation**	**3** ^**rd**^ **generation**	**4** ^**th**^ **generation**
ZrO2 (wt%)	93.4 – 94.5	93.4 – 94.5	89.0 – 92.5	89.0 – 94.5
Y2O3 (wt%)	4.5 – 5.6	4.5 – 5.6	< 10	< 10
Al2O3 (wt%)	0.25 1	0.05	< 0.01	0.05 – 0.01
Other oxides (wt%)	≤ 1.0	≤ 1.0	≤ 1.0	≤ 1.0
**Material Properties**				
Biaxial flexural	1000 – 1500	900 – 1300	400 – 1000	550 – 650
strength (MPa)				
KIc (MPa.√m)	5.0	5.0	2.4 – 3.75	2.4 – 5
Translucency parameter (TP)	16 (0.5 mm thickness)	19 (0.5 mm thickness)	32 (0.5 mm thickness)	20 (1.0 mm thickness)
E (GPa)	210	210	210	210
Hardness (GPa)	13.4	12.3	12.9	15
**Applications**				
Anterior		x	x	x
Posterior	x	x		x
Crown	x	x	x	x
Bridge up to 3	x	x		x
Full bridge	x	x		
Substructure	x	x		
Inlay/onlay			x	
Veneer			x	x

Due to the large selection of zirconia materials, it is difficult for dentists to evaluate and choose the most suitable generation for each specific treatment case. When it comes to the 3^rd^ and 4^th^ generations, the scientific data comparing each other is scarce, mainly because the multilayer system is the latest released on the market. Aiming to fulfill this gap, Jerman, et al.^117^ (2021) evaluated the translucency, biaxial flexural strength, and K_Ic_ of 3Y-TZP, 4Y-PSZ, and 5Y-PSZ materials. For this study, four different non-shaded zirconia, 3Y-TZP (opaque), 3Y-TZP (translucent), 4Y-PSZ (extra translucent), and 5Y-PSZ (ultra-translucent), were evaluated. All samples were sintered according to the manufacturer’s recommendation at 1450 °C for 2 h. The translucency measurement was conducted by a UV–vis-spectrophotometer, the biaxial flexural strength was measured via the piston-on-three balls method, and the K_Ic_ were measured by CNB technique. The results showed that 5Y-PSZ and 4Y-PSZ were more translucent than 3Y-TZP-t and 3Y-TZP-o, with the respective values 34, 33, 30, and 26%. This was due to a variation in composition that included lower Al_2_O_3_ amount and increased Y_2_O_3_ content. For flexural strength, 5Y-PSZ had significantly lower values than the other materials, presenting 259 N/mm^2^ compared to 706 N/mm^2^ of 3Y-TZP-t. This was explained by the material’s lower ability for toughening transformation due to the lower amount of t-phase. Results for K_Ic_ revealed lower values for 4Y-PSZ (3.7 MPa√m) and 5Y-PSZ (2.7 MPa√m) when compared with both 3Y-TZP, which presented very similar results (4.3 MPa√m). This occurred due to lower t → m transformation ability caused by higher Y_2_O_3_ content. Despite the lower values of the material containing higher amounts of Y_2_O_3_, this study allows us to conclude that both materials are suitable for oral restoration. According to the standard DIN EN ISO 6872, 4Y-TZP is adequate for three-unit bridges and molar restorations; and 5Y-PSZ can be used for monolithic restorations, for single crowns in anterior and posterior regions, and three-unit bridges in the anterior region.

The 4^th^ generation of zirconia has been studied to fully realize the range of indications and clinical advantages of this new material. The multilayered zirconia design aims to mimic the natural teeth aspect that changes gradually in the shade. The incisal area of a crown is more translucent, growing in chroma and opacity towards the gingival region. In this context, Kolakarnprasert, et al.^118^ (2019) studied the composition, microstructure, and translucency of a multilayered design composed of three different grades of ZrO_2_: ultra-translucent (UT), super translucent (ST), and multilayered (ML). These three types of material were used to design a multilayered block, in which each layer represented the enamel, transition layer 1, transition layer 2, and dentin. The whole block was sectioned to obtain samples from each layer and then subjected to sintering thermal treatment at 1500 – 1550 °C for ٢ h. For different materials and their layers, the chemical composition, phase fractions, and microstructure were determined by X-ray fluorescence, X-ray diffraction, and field emission scanning electron microscopy. Additionally, their resistance to LTD and translucency properties were characterized. The results revealed no significant differences amongst layers, but the three materials were very distinct. UT with ~75 wt% cubic content and a 4.0 µm average grain size, ST with 65 wt% cubic content and a 2.81 µm average grain size, and ML with 50 wt% cubic content and a 0.63 µm average grain size. After water aging at 120 °C for 12 h, more excellent monoclinic content was found in ML. UT and ST did not show detectable monoclinic phase. The translucency was similar among layers and between UT and ST, which were superior to ML. Finally, for each multilayered zirconia grade, the layers only differed in pigment types and contents, yielding remarkably natural shade gradients. Also, despite the compositional difference between ST and UT, both materials showed similar translucencies.

**Figure 3:**
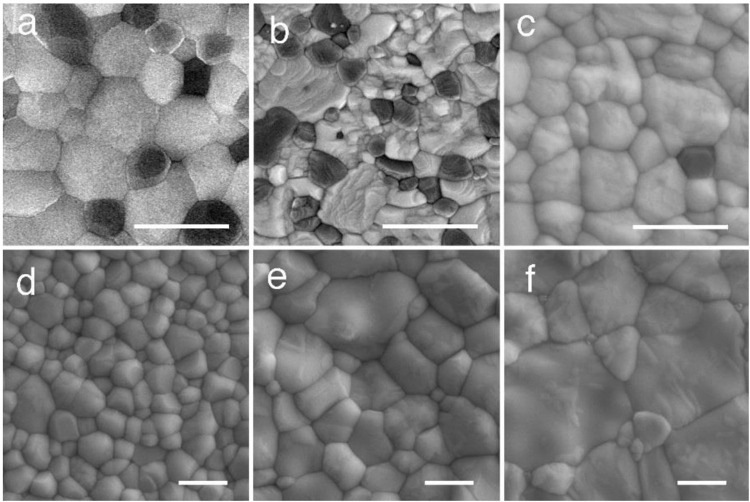
SEM photograph of dental zirconia. (a) ATZ; (b) NanoZR; (c) 3Y-TZP 1st generation; (d) 3Y-TZP 2nd generation; (e) 4Y-PSZ 3rd generation; and (f) 5Y-PSZ 3rd generation. White lines indicating 1 µm scale120; Attribution 4.0 International (CC BY 4.0)

Alumina-toughened zirconia (ATZ) and ceria-stabilized zirconia/alumina nanocomposite (NanoZR) are other types of zirconia-based ceramics widely used in Dentistry for implant fixtures. ATZ comprises 10 – 20% disperse alumina phase in a 3Y-TZP zirconia matrix. This combination brings together the advantageous properties of both ceramics resulting in a material with improved flexural strength, fracture toughness, and LTD resistance.^119^ NanoZR material comprises a 10 mol% Ce-TZP matrix with 30 vol.% of alumina. It presents an interpenetrated intragranular nanostructure in which either nanosized Ce-TZP or alumina particles are located within submicron-sized alumina or Ce-TZP grains, respectively. Besides presenting improved flexural strength, fracture toughness, and LTD resistance, the fatigue of NanoZR shows double the numerical value of 3Y-TZP.^120 , 121^ The different faces of zirconia, and its development evolution, presented different microstructures, as seen in Figure 3 .

ATZ may be successfully functional as an abutment for implant-supported restorations in the long term. Studies showed that ATZ abutment might be clinically successful in implant-supported restorations at both the anterior and posterior regions. The 5-year survival rate of the abutments was more than 95%, regardless of the type of prosthesis.^122^ When prolonging the time for an estimated cumulative 10-year survival rate of the restorations, the result was 94.1% for the FDP prosthesis. These numbers confirmed the successful application of ATZ abutments.^123^

Given the composition evolution of zirconia, we recommend for future investigations to study the latest version of this dental ceramic. In addition to the effects of the composition on the optical properties, it is necessary to evaluate the mechanical properties and the interaction of each layer. Future studies should focus on simulating the oral environment conditions to predict the mechanical performance and durability of the material in addition to clinical monitoring.

## Glass and ceramics

### Glass-Infiltrated Ceramics

The glass-infiltrated ceramics occupied, for some time, an intermediate place between silica-based and polycrystalline ceramics, in terms of mechanical properties and aesthetics. High-strength core ceramics, like zirconia and alumina, could be made more mechanically resistant by glass infiltration with the slip-casting technique. Historically, the main versions of glass-infiltrated ceramic materials were alumina, alumina and magnesium, and alumina and zirconia, which demanded porcelain layering since they were too opaque.^124 , 125^ However, such infiltrated ceramics were progressively discontinued due to the increasing usage of polycrystalline ceramics such as alumina and zirconia.^126^

The ceramic mechanical properties could be improved by the glass infiltration technique since it increases the density of the material. This generates compressive residual stress on the ceramic surface and suppresses fine surface cracks produced by manufacturing processes like sintering and machining. The preform ceramic needs to present a skeleton framework with open, continuous porosity and sufficient rigidity to achieve a homogenous infiltration. This preform is obtained by pre-sintering, and the open pores enable the material to take in and withstand the capillary effect of molten glass infiltration.^127^ The In-Ceram alumina structure was composed of 82% of Al_2_O_3_, 12% of La_2_O_3_, 4.5% of SiO_2_, 0.8% of CaO, and 0.7% of other oxides^128^ ; whereas the alumina and magnesium contained spinel oxide (MgAl_2_O_4_); and the alumina and zirconia were a modification of the original In-Ceram Alumina system, with an addition of 35% partially stabilized zirconium oxide to the slip composition to strengthen the ceramic.^129^

The glass-ceramic systems In-Ceram Alumina and In-Ceram Spinell were used for single restorations, inlay and onlay, and anterior fixed restorations, not being recommended for posterior applications. In-Ceram Zirconia was better suited this purpose, and was developed for a posterior fixed partial prosthesis.^130^ These glass-infiltrated materials are no longer available in the market.

## Functionally graded materials

Functionally Graded Materials (FGMs) are a type of glass-infiltrated ceramic. The FGMs are described by a gradual change from layer to layer, promoting gradual variations in the macroscopic properties of the material.^131 , 132^ This creates a gradient in composition along with the ceramics. Elastic modulus, for example, gradually increases since glass has a lower modulus than the general dental ceramics used for restoration. At the surface, the elastic modulus is closer to the modulus of glass; as it goes deeper into the bulk, the elastic modulus gradually increases until it reaches the modulus of the ceramic.^133^ The gradual change in composition tends to optimize the material performance across the whole volume since it eliminates the well-defined interface between materials, where failure is often initiated. This is the reason why functionally graded infiltrated glass ceramics are reported to exhibit superior mechanical properties compared to monolithic structures.^131^

In the FGM process, a previously prepared glass penetrates the grain boundaries of the monolithic ceramic driven by capillary pressure. The resulting structure consists of a thin, outer surface glass layer about 15 µm thick, followed by a graded glass/monolithic zirconia layer, about 120 µm thick, and a dense homogeneous ceramic core. The outer layer is composed only of glass with a consecutive graded interface of around 45 vol% of glass. This technique allows superior aesthetic aspects since different shades of glass can be used.^134^

The main ceramic framework used for glass infiltration is alumina (Al_2_O_3_), ZTA, and zirconia 3Y-TZP due to their high flexural strength (600 MPa, 700 MPa, and 1000 MPa, respectively) and K_Ic_ (3.1 MPa.√m, 4.8 MPa.√m, 5 MPa.√m, respectively).^135 - 137^ For glass infiltration, the main compounds include oxides of La, Si, B, Ba, Al, Zr, Y, Ti, Ca, K, and Na.^138 , 139^ Bioglass 45S5 is bioactive, biocompatible, and capable of producing strong chemical bonds with zirconia surfaces. However, this type of glass presents an incompatible CTE with zirconia resulting in an unfeasible material for this application.^140^

The influence of surface treatments for ceramics is a topic of ongoing investigation. In addition to increasing the mechanical properties, the glass-infiltration method can modify the optical properties of the material. Considering ceramic restorations – such as feldspathic ceramic, leucite-reinforced feldspathic ceramic, polymer-infiltrated ceramic, glass-infiltrated ceramic, and some restoration based on resin composite – a study was published illustrating the relation between mechanical and optical properties of these materials. Regarding flexural strength, the glass-infiltrated ceramic presented superior flexural strength (600 MPa) compared to the other material. In contrast, the infiltrated configuration (polymer and ceramic) presented lower translucency compared to the other ceramic types.^141^

Although some commercial zirconia has been used to manufacture monolithic crowns, some of these materials present poor translucency due to their high refractive index, low absorption coefficient, and high opacity in the visible and infrared electromagnetic spectra. In this context, Volpato, et al.^142^ (2019) evaluated the color and translucency of glass-infiltrated zirconia based on the concept of functionally graded materials. The authors produced samples of Y-TZP and divided them into three groups: no treatment (NT), immersed in a coloring liquid (A2), and immersed in a fluorescent liquid (F). Afterward, half of the samples from each group were treated with glass infiltration based on the FGMs concept. A commercial glass (VITA In-Ceram S1; VITA Zahnfabrik) was applied using the protocol of immersing or not the samples in the liquids, followed by a thermal cycle that involved heating, cooling, and sintering steps. A spectrophotometer measured the initial color of the samples in the reflectance mode on an absolute white background. The translucency parameter (TP) was obtained on a black background, and measurements to obtain the contrast ratio (CR) were made on the white and black backgrounds by the CIEXYZ system. Next, the samples were subjected to an accelerated aging protocol for 4 hours in an autoclave at 134 °C. Then, another measurement of color, TP, and CR were accomplished. The results showed that the color and translucency of the zirconia were altered after glass infiltration in almost all the tested groups, especially the color group A2. The research pointed out that the glass increased the lightness of the zirconia, whereas aging treatment affected its chroma. In turn, no perceptible differences were found in the untreated group, even after aging. A decrease in translucency was observed after the use of coloring and fluorescent liquids, as well as after infiltration. This suggests that the presence of glass within the zirconia microstructure probably prevented light transmission. This study concluded that glass infiltration influences the optical properties of the zirconia. However, further studies are needed to verify whether the fluorescence obtained with laboratory procedures is similar to the phenomenon in natural teeth and whether the coloration obtained with immersion liquids is constant and reproducible.

Engaged with this type of dental ceramic restoration, Arnesano, et al.^143^ (2020) studied the fabrication of thermoplastic filaments of glass-infiltrated alumina ceramics for 3D printing by fused deposition modeling (FDM). The 1.75 mm diameter filaments were produced by melting extrusion using Al_2_O_3_ powder, a multi-component organic system composed of low-density polyethylene (LDPE), polylactic acid (PLA), paraffinic wax, and polyethylene grafted maleic anhydride (PEgMA). Two types of filaments were produced: one containing 50 vol% of Al_2_O_3_ and the other 57 vol%. The specimens were printed and subjected to a chemical dewax. After that, a pre-sintering process was performed at 1150 °C for 2 h. A lanthanum-based glass powder (VITA In-Ceram^®^) was used to infiltrate the samples by applying a thin layer of glass frits on the porous surface at 1120 °C. The glass-infiltrated samples, characterized by XRD, showed no peak shifts associated with residual compression stress. This was regarding the proximity of glass and alumina CTE values ( $8.1\times 10^{-6}/{}^{\circ }C$ ). The analysis by Scanning Electron Microscopy with Energy Dispersive X-ray Analysis (SEM-EDX) revealed a residual porosity, after infiltration, which was measured using Hg porosimetry resulting in 3% for 50 vol% of Al_2_O_3_ and 3.5% for samples with 57 vol% of Al_2_O_3_. From the three-point bend test results, it was evident that glass infiltration greatly improves the mechanical resistance of alumina. Glass infiltrated samples with higher solid load (57 vol%) showed ~10% higher strength (264 MPa) compared to lower alumina loading (238 MPa). Based on these results, the authors concluded that combining FDM with glass-infiltrated ceramic can be a new approach for all-ceramic dental prostheses fabrication in near-net shape. Further studies are needed to understand the influence of the printing parameters and solid load on mechanical properties after infiltration to improve strength and accuracy.

The benefits of glass-infiltrated ceramic highlighted in the previous sections have contributed to the ever-improving standard of teeth restoration. This material can improve many properties, such as mechanical, optical, aesthetics, and tribological. Thus, glass-infiltrated ceramic is a good choice for dental prostheses and a solution to the high chipping rates associated with porcelain-veneered crowns.^9^

## Conclusions

This review pointed out the last development of restorative ceramic materials, including all-ceramic and metal-ceramic types. Their applications show the current advantages and limitations yet to overcome. As seen from PFM to multilayer zirconia, no material can fulfill all the needs existing in clinical situations. The current need is to reach a balance between good mechanical properties and high-quality aesthetic finishing aiming to mimic the optical aspect of natural teeth. Also, considering the novelty of zirconia Y-TZP and ATZ, the reliability of these materials requires further laboratory and clinical investigation.
